# Clinical Remarks upon Surgical Cases in the Buffalo General Hospital—Removal of Both Ovaries—Death

**Published:** 1867-12

**Authors:** J. F. Miner


ART. III—*Clinical Remarks upon Surgical Cases in the Buffalo General Hospital—Removal of both Ovaries—Death.* BY J. F. MINER, M. D.

The present interesting case, in an intelligent and highly respectable private patient, who has consented to your being invited to be present, will be explained by Prof. White, who has invited you to see an operation which I understand has been fully described and explained in his lecture this morning, and who has kindly consented to be present and assist in the operation. He will also make such further explanations as he may deem proper, and describe the processes found necessary to adopt in this particular case. The description of the peculiar features present and worthy of especial notice, will be given by him. I shall also be assisted by Drs. Eastman, Lothrop, Wyckoff, and C. Nichell, and by a number of professional friends, both from the city and neighboring towns, among whom I may mention Drs. Skinner, Eddy, Mixer, Loomis, Brown, E. Barnes, Shaw, Smith, Potter, Eddy, of Lewiston, Diehl, Janson, Daggett and others, whose names I am now unable to call.

PROF. WHITE, after expressing his pleasure in having opportunity to take his class from the lecture hall directly to the operating room, and thanking Dr. Miner for such rare opportunity, remarked nearly as follows:

*Gentlemen:*—The patient now presented before you, Mrs. B——, is thirty years old, has enjoyed tolerable health during her whole life until within the last five years. She is the mother of one child, and appears free from disease except what is situated in the abdominal cavity and the effects it may have produced elsewhere. The abdomen is immensely distended, we have no doubt, from serous effusion—that there is *ascites* in connection with and complicating disease of the ovary. Such immense distension of the abdomen is uncommon, and when present in so great degree, is an unfavorable symptom. To determine the existence of adhesion in this case is certainly impossible; in almost all cases it is difficult and uncertain.

The patient has been suffering with the condition you observe, relieved occasionally by tapping, for the last three years, and come at length to desire the chances, whatever they may be, of ovariotomy. As I have this morning told you, statistics thus far, will not justify expectation of a greater than about 50 per cent. of recoveries; making it an operation not to be lightly or unhesitatingly adopted. Recent improvements in making the operation it is believed will raise the per cent. of recoveries, and if only the most favorable cases are selected and these placed in the hands of judicious operators, a very large per cent. of recoveries could be reasonably expected. But the condition of this disease is such that without removal there is *no* hope of recovery; if there is any chance, however small, patients frequently insist upon their right to it, and the humane surgeon cannot refuse operation, though his judgment may decide the chances very unfavorable; he must do his duty and leave the results; sometimes most unfavorable cases unexpectedly recover.

The first procedure in the operation, after every preparation has been made, and the patient has been brought fully under the anæsthetic, is the *exploratory incision;* this, you will observe, has been most carefully and skillfully made, and you can now see through the transparent walls of the peritoneum the clear serum which fills the cavity of the abdomen in so great degree. A large trocar is introduced and something near forty-five pounds of serum removed. This brings the ovarian tumor into distinct view, and the incision is extended through the linea alba, five or six inches. Upon examination we find that it is strongly adherent throughout its whole extent to the peritoneum, the connection being by strong, firm bands and broad fascia like expansions, of similar material as the bands, being prolongations, either from the peritoneum to the tumor, or *vice versa.* These are separated at the tumor, carefully broken or peeled off from its surface, and after great care and effort the pedicle of the tumor is reached, which is large, short, and contains several arteries of large size. Upon examination, also, the other ovary is diseased, consists of a sac which is filled with a steatomatous material, intimately mixed with hair. These sacs are rare, and have given rise to a great deal of speculation as to their origin, some regarding it as evidence of former extra uterine pregnancy; others believing the hair a growth from the walls of the sac. The small tumor which will weigh three or four ounces, is readily removed by applying the clamp and dividing the pedicle with the actual cautery; no hemorrhage follows its division, and its removal is quite satisfactorily obtained. The large tumor, which has been allowed to remain until after the smaller is removed, is treated in similar manner; but the vessels are large, and though subjected to ample and satisfactory cauterization, when the clamp is removed, immediately show that the hemorrhage cannot be thus arrested. There are so many of these large vessels that in this case it is deemed best to apply double ligature, tying both ways upon the pedicle and bringing it out at the lower angle of the incision. All the fluid from the tumor and the bloody serum from the torn adhesions which has accumulated, is now carefully sponged from the cavity of the pelvis, and the incision closed by deep silver sutures extending to the peritoneum, and a few superficial ones to more completely adapt the margins. Adhesive strips are applied after the method already described, extending completely around the body so as to insure rest and support. The patient is removed to her room, and has well borne, so far as can now be seen, the fearful operation of removal of both ovaries, and ascitic fluid sufficient to make in the aggregate sixty-one pounds.

Upon opening into the interior of the principal tumor, we find another, much larger sac than before, containing similar matter, and completely filled with long, coarse hair, some of which can be shown to have grown from the cyst wall, certainly to be closely adherent to it at one extremity.

Whatever may be the termination of this interesting and instructive case, the care, skill and judgment in its removal by Dr. Miner could not be surpassed, and it offers the only possible ground of hope which such disease affords, though it seems that in this case recovery cannot be very confidently expected.

NOTE.—The first and second days after operation the patient was remarkably cheerful, hopeful and comfortable, vomiting being the only unpleasant symptom. The third day signs of depression were present, and neither food or medicine retained upon the stomach. Death took place on the morning of the fourth day, from exhaustion.

